# Two new species in the family Axinellidae (Porifera, Demospongiae) from British Columbia and adjacent waters

**DOI:** 10.3897/zookeys.338.5535

**Published:** 2013-10-02

**Authors:** William C. Austin, Bruce S. Ott, Henry M. Reiswig, Paula Romagosa, Neil G. McDaniel

**Affiliations:** 1 Khoyatan Marine Laboratory, 9245 Hartfell Road, North Saanich, British Columbia, Canada V8L 5G5; 2 Biology Department, University of Victoria and Royal British Columbia Museum, P.O. Box 3020 Stn CSC, Victoria, British Columbia, Canada V8W 3N5

**Keywords:** Porifera, Demospongiae, Halichondrida, Axinellidae, northeast Pacific, *Auletta krautteri*, *Dragmacidon kishinensis*

## Abstract

Two new species of Demospongiae are described for British Columbia and adjacent waters in the family Axinellidae, *Auletta krautteri* **sp. n.** and *Dragmacidon kishinensis* **sp. n.** They represent range extensions for both of these genera. Both are fairly commonly encountered, *Auletta krautteri* below diving depths (87 to at least 300 m) and *Dragmacidon kishinensis* in shallow water (intertidal to 30 m). We propose an amended genus diagnosis for *Auletta* to account for the variability among species in principal spicules that form the ascending tracts to be either oxeas, styles or strongyles rather than just oxeas.

## Introduction

A brief history of surveys and publications from 1878 to 1966 including sponges in British Columbia is presented in Austin et al. (in prep.). Of the approximately 200 demosponge species recorded for this region (Austin 1985, Austin et al. 2012), six species were regarded as members of the family Axinellidae (Austin 1985, Austin and Ott 1987). Three of these have now been removed from this family. *Syringella amphispicula* de Laubenfels, 1961 was recently shown to belong to the genus *Homaxinella* and hence to the family Suberitidae (Austin et al. in prep.). *Stylissa stipitata* de Laubenfels, 1961 is not a *Stylissa* and not an axinellid; It is considered to be a synonym of *Semisuberites cribrosa* (Micklucho-Maclay, 1870) by van Soest et al. (2012). Specimens identified by us as *Phakettia* sp. aff. *beringensis* we now consider to be *Semisuberites cribrosa*. Finally, a specimen identified as *Axinella* sp. has been lost. We defer any formal description until we have more material. It has been seen twice from a submersible at 100 m. The remaining two species, both new, are described in this paper: *Auletta krautteri* sp. n. and *Dragmacidon kishinensis* sp. n.

Alvarez and Hooper (2002) provided a discussion of the history, definition and scope of the Axinellidae. They reviewed the literature and concluded that among 92 nominal genera, only 10 were valid (*Auletta*, *Axinella*, *Cymbastela*, *Dragmacidon*, *Dragmaxia*, *Pararhaphoxya*, *Phakellia*, *Phycopsis*, *Ptilocaulis*, and *Reniochalina*).

## Materials and methods

Specimens housed in the Khoyatan Marine Laboratory museum (KML) were largely preserved and maintained in 70% isopropyl alcohol; some are dried. KML specimens were collected either by hand in the intertidal, by SCUBA in the shallow subtidal, or by dredge, submersible (*PISCES IV, DELTA*) or ROV (*ROPOS*) in deeper water.

Collections were examined for axinellids from the NE Pacific at Royal BC Museum, California Academy of Sciences, and the Canadian Museum of Nature. In some cases material was brought back to KML for more detailed study.

Under material examined, for each lot we report: institution accession number, station number, location, latitude and longitude, depth, date of collection, collector, and number of specimens. Where geographic location but not latitude and longitude was recorded, we include them following the abbreviation for approximate: ca.. We endeavoured to track down missing data but were not always successful. Sources for obtaining geographic coordinates include: Google Earth, Sailing directions BC Coast (North & South Portion), United States Coast Pilots: Pacific Coast #8, southern Alaska, and #7, BC Geographical Names Information System, and Canadian Hydrographic Service charts.

Where colour photographs were taken, in situ scale bars are approximate. Photos taken together with specimens are assigned the same station number and accession number as those specimens.

Thick sections of specimens were made by excising approx. one cm^3^ surface blocks, dehydrating in ethanol and embedding these in 68°C melting point histological paraffin. After cooling to room temperature, the blocks were trimmed to either vertical or tangential orientation and re-warmed to 40°C for one hour to prevent cracking during sectioning. Warmed tissue blocks were set into a guiding jig and sectioned by hand with a straight razor at varied, but only marginally controllable thicknesses of between 0.1 and 1.0 mm. The best sections were de-paraffinized in xylene and mounted on microscope slides in Canada balsam for observation, measurement and photography.

Tissue-free spicule preparations were made by dissolving small pieces of sponge in sodium hypochlorite. For each spicule type we measured, using a compound microscope, the diameter or length and width of 50 spicules (unless noted otherwise by N=). We scanned microscope fields for spicules of variable sizes, but ignored obviously ontogenetically young spicules in determining size ranges. We list spicule dimensions as three numbers, the minimum, mean and maximum, e.g., 200–(250)–300. All measurements are in micrometers (μm). For scanning electron microscopy (SEM), cleaned spicules were either deposited onto membrane filters that were then taped to stubs, or deposited directly on double-sided tape attached to stubs. Preparations were coated with gold-palladium and viewed either in a Hitachi S-3500N SEM at the University of Victoria, or in one case an ETEC Biosem at Simon Fraser University.

Holotypes have been deposited in the Royal British Columbia Museum, Victoria, BC, Canada and paratypes have been deposited in the Canadian Museum of Nature, Ottawa, Ontario.

Taxa including families, genera and species are arranged alphabetically. Systematic hierarchy follows Hooper and Van Soest 2002. Abbreviations used in the text ordered alphabetically are: approx.=approximate; BC=British Columbia; CASIZ=California Academy of Sciences, Invertebrate Zoology, San Francisco, California, U.S.A.; CMNI=Canadian Museum of Nature, Ottawa, Ontario, Canada; coll.=collector; FRB=Fisheries Research Board of Canada, Ottawa, Ontario, Canada; ID=identified; KML=Khoyatan Marine Laboratory, lat.=latitude; long.=longitude; North Saanich, BC, Canada; PBS=Pacific Biological Station, Nanaimo, BC, Canada; PEI=Pacific Environment Institute, Fisheries and Oceans Canada, West Vancouver, BC, Canada; RBCM=Royal British Columbia Museum, Victoria, BC, Canada; Str.=strait.

## Descriptions

### Genus *Auletta* Schmidt, 1870

**Systematics**

Phylum Porifera

Class Demospongiae

Order Halichondrida

Family Axinellidae Carter, 1875

Genus *Auletta* Schmidt, 1870

**Genus diagnosis.** Tubular, erect on peduncle or narrow base. Surface smooth or tuberculated with choanosomal spicules projecting slightly; ectosome without specialised skeleton. Choanosomal skeleton plumoreticulate, with longitudinally strongyle or sinuous oxea tracts, connected by single styles or plumose tracts of styles; masses of sinuous strongyles reinforcing the stem and may reinforce the inner tube wall. Megascleres sinuous strongyles or oxeas, always coring main spicule tracts and inner tube walls; styles and or oxeas, plumo-echinating and connecting main tracts. Microscleres absent.

(Amended from Alvarez and Hooper 2002)

#### Auletta krautteri sp. n.

http://zoobank.org/B1D4806D-AE5D-4D4E-8DDC-6B5E2685BB76

http://species-id.net/wiki/Auletta_krautteri

Fig. 1

##### Etymology.

Named after Dr. Manfred Krautter who organized a dive program in the submersible *DELTA* on sponge bioherms and collected the holotype.

##### Material examined.

Holotype: RBCM 013-00114-001; KML1105 KML Sta. 71/99 Hecate Strait, BC, (52°26.4'N, 129°40.0'W), 215 m depth, July 18, 1999, coll. M. Krautter, 1 specimen. Paratype: CMNI 2013-0001, KML1106, west of Dixon Entrance, BC, (54°370'N, 133°55.0'W), 229 m depth, 1 specimen.

##### Other material.

KML1106, PBS 65-77, west of Dixon Entrance, BC, (54°37 0'N, 133°55.0'W), 229 m depth, 21 specimens; KML1108, PBS JWS-132, Queen Charlotte Sound, BC, (51°22.5'N, 129°13.5'W), Feb. 3, 1965, 16 specimens; KML1107, KML Sta. 171/76, West of Flamingo Inlet, BC, (52°09.8'N, 131°23.8'W), 200 m depth, Aug. 31, 1976, coll. W.C. Austin, 3 specimens; KML1109, PBS 71-47, off Dixon Entrance, BC, (54°30.2'N, 135°53.3'W), 256 m depth, 3 specimens; KML1105, KML Sta. 71/99, Hecate Strait, BC, (51°21.5'N, 129°13.5'W), 183 m depth; CASIZ 020231, NODC 366501, Gulf of Alaska, (59°2.0'N, 141°3.6'W), 348 m depth, 2 specimens; KML1108, PBS 981-60, Dixon Entrance, BC, (54°N, 132°W), 128 m depth; CMN 1900-86, Forester I., Alaska, (54°48'N, 133°36'W), depth no data, coll. W. Van Vliet; CMN 1900-89, sta. LM 43, Tasu, Queen Charlotte Islands, BC, (52°45'N, 132°06'W), depth no data, coll. L. Marhue; CMN 1900 sta. JWS-93, Forester I., Alaska, (54°48'N, 133°36'W), depth no data, coll. J.W. Scogoen; CMN 1900-91, W. of Queen Charlotte Islands, BC, (53°N, 132°W), depth no data, coll. W. Van Vliet; CMN 1900-93, sta. FRB 66 221 m depth, Forester I, Alaska, (54°48'N, 133°36'W), depth no data; CMN 1900-94, sta. LM-43, Tasu, Queen Charlotte I., BC, (52°45'N, 132°06'W), depth no data, coll. L. Marhue; CMN 1900-96, sta. FRB 66-2-6, off Sitka, Alaska, (57°02.3'N, 135°20.3'W), depth no data, coll. W. Van Vliet.

##### Description.

**Figure 1.**
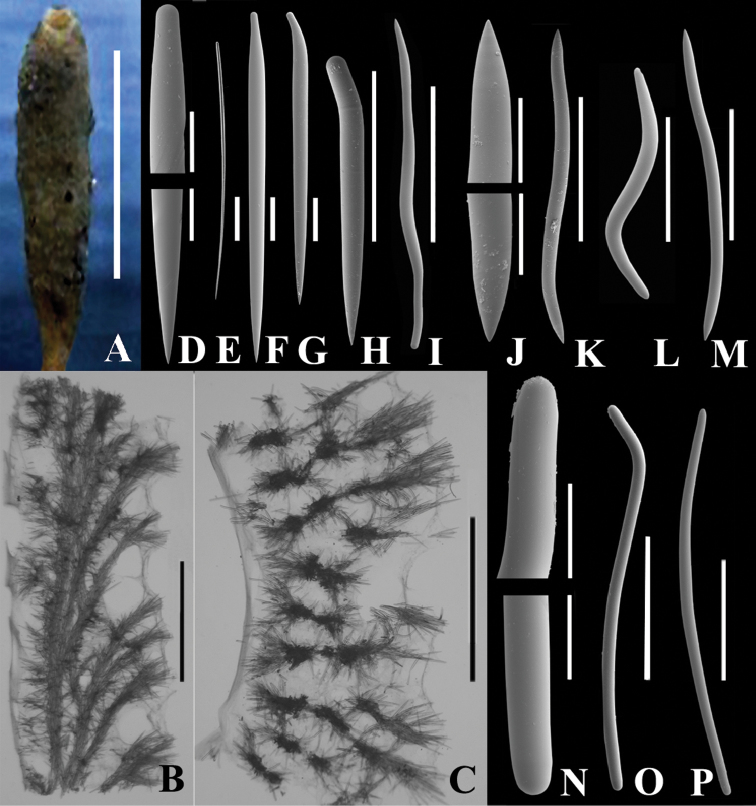
*Auletta krautteri* sp. n. **A** Fresh specimen, KML1107, KML Sta. 171/76, West of Flamingo Inlet, BC, scale bar 5 cm **B** KML1105, vertical longitudinal section, scale bar 3 mm **C** KML1105, cross section, scale bar 3 mm; D–O. KML1105, spicules **D** ends of style, scale bar 50 µm **E** style associated with osculum (under light microscope), scale bar 100 µm **F–I** various forms of styles **F** scale bar 100 µm; G. scale bar 100 µm **H** scale bar 200 µm **I** scale bar 300 µm **J** ends of oxea, scale bar 50 µm **K–M** various forms of oxeas **K** scale bar 300 µm **L** scale bar 200 µm **M** scale bar 300 µm **N** ends of strongyle, scale bar 50 µm **O, P** two forms of strongyles, scale bar 300 µm.

***Macroscopic features*.** Erect, stalked tubes typically single (Fig. 1A), occasionally branched (2 to 3 tubes on a common base); branched forms uncommon. Overall height 5–13 cm, width of tubes 0.7–2 cm. Stalk comprises up to one third of overall height. A single 2–8 mm diameter osculum at the tube apex leads into an atrial cavity extending the length of the tube and into the stalk where the tube diameter is restricted. Wall thickness of the tube 5–10 mm. Surface felt-like to touch. Smooth inner wall penetrated by a series of elongate openings. Consistency compressible but firm and tough. Colour in life reddish-brown; grey or cream in alcohol. Specimens collected in 1965 contained oocytes 130 to 150 µm diameter.

*Microscopic features*. Skeletal architecture simple, composed of one to three multi-spicule tracts oriented parallel to and lining the atrial cavity, which is relatively smooth as a result (Fig. 1B). Single, or multispicular tracts branch from this longitudinal tract approximately at right angles and project to the outer surface. The branches also form short brushes, and where each branch penetrates the surface, the terminal brush forms a tuft to produce a hispid appearance (Fig. 1C).

Each tract varies from 150–400 µm in diameter. Ascending tracts are composed primarily of straight and curved styles, and secondarily of sinuous oxeas, curved oxeas and occasional sinuous strongyles (Fig. 1O, P). Straight styles or styles curved near the base form the exterior tips of ascending fibres and curved, bent or sinuous oxeas and styles form cross tract links.

The multi-spicule tracts of the atrial cavity are 500–700 µm diameter and composed of bundles of 10 to 15 spicules cemented by spongin Ascending tracts composed of fewer, typically 5 or 6, spicules in a bundle cemented by spongin. Atrial tracts composed primarily of sinuous oxeas, secondarily of curved and straight styles; occasionally sinuous strongyles, sinuous styles, and curved oxeas located in axial tracts.

Ectosome surface forms a reticulation in the areas with pores where it is elevated about 2 mm above the general surface. Easily detachable aspicular membranes are present on dermal surface stretched between spicule tracts, and on atrial surface below the longitudinal spicule tracts (Fig. 1B, C). The choanosome occupies the space between the detachable membranes and is distinguished by radial orientation of the spicule tracts, and by the somewhat different proportion of spicules, which is quite variable among different specimens.

Oscula may be ringed by long, straight styles singly or in tufts. Fringe may be absent, but if present, extends 100–300 µm beyond the osculum.

Stalk is denser than the tube, not hollow except near the tube base, and packed with branching and anastomosing multi-spicule tracts, forming a dense reticulation of two to ten or more spicules to a bundle cemented by spongin. Stalk tracts 100–400 µm diameter. Primary spicules sinuous strongyles which serve to reinforce the stem. The proportion of other stalk spicules is quite variable with sinuous oxeas, bent and curved and straight styles being variably the next most abundant. Sinuous styles and curved or bent oxeas are uncommon.

*Spicules*. Spicule types include straight (Fig. 1F) and bent (Fig. 1H) styles of the multi-spicule tracts; long, straight styles of the oscular fringe (Fig. 1E) and proximate area; sinuous (Fig. 1K, L), curved or bent (Fig. 1M) oxeas, and sinuous strongyles (Fig. 1O, P). Occasionally sinuous oxeas occur that are rounded on one end forming sinuous styles. These latter were enumerated separately to give a qualitative idea of their abundance.

Longer styles often have a reduced diameter at the head comparable to mycalostyles. Oxeas are often anisometric. Both oxeas and styles occasionally have mucronate or rounded apices. Oxeas and strongyles may occasionally be centrotylote.

Five specimens were examined in detail (Table 1).

**Table 1.** Comparison of spicules in *Auletta krautteri* specimens examined in detail.

**ID**	**Location**	**Latitude, Longitude**		**Depth (m)**	**Length (µm)**					
					**Straight Style**	**Curved Style**	**Sinuous Oxea**	**Curved oxeas**	**Sinuous Strongyle**	**Oscular Styles**
KML1105	Hecate Strait, BC	52°26.4'N, 129°40.0'W		215	270–1350	240–880	440–1300	330–1070	420–1595	none
Spicule Abundance	Many straight and curved styles, and sinuous oxeas throughout. Strongyles abundant in stem, rare in atriosome, uncommon in ectosome. Curved oxeas uncommon in stem. Bent and curved oxeas uncommon to moderately common.									
KML1106	Dixon Entrance, BC	54°37.0'N, 133°55.0'W		229	320–1280	220–990	380–1274	220–880	250–1430	none
Spicule Abundance	Styles and sinuous oxeas abundant in ectosome and atriosome, uncommon to moderately common in stem. Strongyles abundant in stem, uncommon in ectosome and rare in atriosome. Bent and curved oxeas uncommon to rare.									
KML1108	Goose Bank, QC Sound BC	51°30.0'N, 128°0.0'W		92	111–1300	119–940	310–1100	250–880	300–1150	970–2375
Spicule Abundance	Straight styles abundant in ectosome, common in atriosome, uncommon in stem; bent styles abundant throughout. Sinuous oxeas common throughout. Strongyles abundant in stem, uncommon in atriosome, rare in ectosome. Bent and curved oxeas moderately common except rare in stem.									
KML1109	Dixon Entrance, BC	54°30.2'N, 133°53.3'W		256	250–1500	230–720	105–1250	123–920	440–1120	680–2450
Spicule Abundance	Styles and sinuous oxeas abundant throughout. Sinuous strongyles abundant in stem, uncommon elsewhere. Bent and curved oxeas rare in stem, common elsewhere.									
KML1107	W of Flamingo Inlet, BC	52°89.8'N, 131°23.8'W		200	325–926	220–790	325–1005	276–768	286–1281	926–1050
Spicule Abundance	Styles and sinuous oxeas abundant in the ectosome and atriosome; uncommon to rare in the stem. Strongyles abundant in the stem, rare in atriosome, uncommon in the ectosome. Bent & curved oxeas rare in stem, uncommon elsewhere.									

##### Remarks.

Evident from the Table 1 above is the relatively large variability in disposition and size of spicules from specimen to specimen.

Our specimens fit the diagnosis for *Auletta* by Alvarez and Hooper (2002) except that the sinuous diacts are primarily oxeas in the tube and strongyles in the stem. Several species of *Auletta* are reportedtohave sinuous oxeas but no strongyles (e.g., *Auletta aurantiaca* Dendy, 1889, *Auletta consimilis* Thiele, 1898, *Auletta pedunculata* Topsent, 1896, *Auletta lyrata* (Esper, 1794)).

The following species are not conspecific with *Auletta krautteri* based on the absence of one or more spicule types.

*Auletta andamensis* Pattanayak, 2006, p. 66 No strongyles

*Auletta aurantiaca* Dendy, 1889, p. 92No strongyles

*Auletta consimilis* Thiele, 1898, p. 55 No strongyles

*Auletta dendrophora* Wilson, 1904, p. 158 No oxeas

*Auletta grantioides* Lévi & Vacelet, 1958, p. 243 No oxeas

*Auletta halicondroides* Thiele, 1898, p. 55 No strongyles

*Auletta lyrata* (Esper, 1794) No strongyles

*Auletta lyrata* var. *brevispiculata* Dendy, 1922 No strongyles

*Auletta pedunculata* Topsent, 1896 No strongyles

*Auletta sessilis* Topsent, 1904 No oxeas

*Auletta sycinularia* Schmidt, 1870 No oxeas

*Auletta tubulosa* (Ridley & Dendy, 1886), p. 482 No oxeas or strongyles

*Auletta tuberosa* Alvarez, Van Soest & Rützler, 1998, forms clusters of tubes which are tuberculate rather than smooth as in *Auletta krautteri*. It does have oxeas, but they are smaller (340–430–530) than in *Auletta krautteri*. *Auletta elongata* Dendy, 1905: external form consists of multiple tubes branching off a single stem rather than single tubes on each stem as in *Auletta krautteri*. The axial skeleton consists of stout fibres with short perpendicular anastomosing branches rather than single to three longitudinal axial fibres with relatively long arching perpendicular fibres that branch but do not anastomose. *Auletta elongata* var. *fruticosa* Dendy, 1916, is similar to *Auletta elongata* except it has smaller spicules.

Two other sponges originally assigned to *Auletta* have been reassigned to other genera: *Auletta elegans* Vosmaer, 1882, is now accepted as *Semisuberites cribrosa* (Miklucho-Maclay, 1870) (van Soest et al. 2005): Barents Sea. *Auletta celebensis* Thiele, 1899 is now accepted as *Stylissa massa* (Carter, 1887) (Van Soest et al., op. cit.): West Pacific.

Stone et al. (2011) briefly described and showed images of a tubular form they identified as *Axinella rugosa* (Bowerbank, 1866) that might be con-specific with *Auletta krautteri*. However, *Axinella rugosa* in the N. Atlantic is described as bushy with irregular branches (van Soest 2013). Lambe (1895) identified four specimens from Chika Island and Unalaska Island as belonging to *Axinella rugosa*. However, Cuenot (1913) argued that these were not *Axinella rugosa* and proposed a new name *Phakellia lambei* Topsent, 1913. Lambe (1895) described his specimens as dividing close to the base into two branches which subdivide above into two lobate expansions.

##### Conclusions.

No described species have a suite of characters matching those of our specimens. We therefore propose that the *Auletta* in British Columbia and Alaska be considered a new species, *Auletta krautteri*. We suggest that the tubular forms recorded by Stone et al. (2011) are likely *Auletta krautteri*.

##### Bathymetric range.

180 to 320 meters depth; 87 to 712 meters depth if include *Axinella rugosa* of Stone et al. (2011).

##### Zoogeographic range.

Gulf of Alaska and south to the southern end of the Queen Charlotte Islands, BC, also central Aleutian Islands if include *Axinella rugosa* of Stone et al. (2011).

##### Ecology.

The sponge is a moderately common dredged species found on rock, gravel or mud substrates. Some individuals have been found with a small red copepod (unidentified) burrowed into the surface. Two individuals examined contained a species of the isopod *Gnathia*, oriented head down. Unidentified gammarid amphipods and unidentified spionid polychaetes have also been observed. Numbers of the crinoid *Florometra serratissima* (A.H. Clark, 1907) were observed clinging to specimens of *Auletta krautteri* in Hecate Strait, BC.

### Genus *Dragmacidon* Hallman, 1917

**Genus diagnosis.** Unbranched, club-shaped, lobate, shrub-like, thickly encrusting or massive habit. Surface with short connules or tubercles; oscules circular, flush or slightly elevated, sometimes with superficial canals leading to opening. Ectosome without specialised skeleton. Choanosomal skeleton plumoreticulate with ascending plumose tracts, anastomosing or interconnected by secondary multispicular tracts; not differentiated into axial or extra-axial regions. Megascleres oxeas and/or styles generally in similar proportions and dimensions. Microscleres, if present, raphids in tightly packed trichodragmas (Alvarez and Hooper 2002)

#### Dragmacidon kishinensis sp. n.

http://zoobank.org/53DA9618-DE0A-47E4-B78F-BB772C1D1400

http://species-id.net/wiki/Dragmacidon_kishinensis

Fig. 2

##### Etymology.

After the ancient First Nation (aboriginal) village site kiix?in (pronounced keeshin) which includes Execution Rock Cave, Barkley Sound, BC, where a specimen was collected in the low intertidal.

##### Material examined.

Holotype: RBCM 013-00115-001, KML1111, PEI 44, Steep I., Discovery Passage, BC, (50°4.94'N, 125°15.35'W), 30 m depth, coll. N. McDaniel, Feb. 26, 1976, 1 specimen & in situ image. Paratype: CMNI 2013–-0002, KML1113, KML 139/80, Limestone I., BC, (ca. 52°55'N, 131°36'W), 3 m depth, coll. W.C. Austin, July 4, 1980, 1 specimen.

**Other material.** KML1112, PEI 130, Copper Cliffs, Discovery Passage, BC, (50°6.40'N, 125°15.35'W), 15 m depth, coll. N. McDaniel, Apr. 16, 1978, 1 specimen; KML1114, PEI 53, Grilse Pt. Texada I, BC, (49°48.03'N, 124°35.79'W), 10 m depth, coll. N. McDaniel, Mar. 13, 1977, 1 specimen; KML1116, PEI 49, Vivian I., BC, (49°50.28'N, 124°41.96'W), 15 m depth, coll. N. McDaniel, Apr. 10, 1976, 1 specimen; KML1121, Sta. no data, Rennell Sound, BC, (ca. 53°24'N, 132°44'W.), depth no data, coll. M. LeBlanc, Apr. 14, 1989, 2 specimens; KML1294, KML 127/76, Execution Rock Cave (48°48.9'N, 125°10.6'W), 0.2 m height, coll. W.C. Austin, July 28, 1976, 1 specimen; RBCM 976-1081, Entrance I., Tasu Sound, BC, (ca. 52°46.04'N, 132°03.55'W), depth no data, coll. P. Lambert, 1976, 1 specimen; RBCM 974-230-3, Brundige Inlet, BC, (ca. 54°37'N, 130°50'W), coll. P. Lambert, June 19, 1974, depth no data.

##### Description.

**Figure 2.**
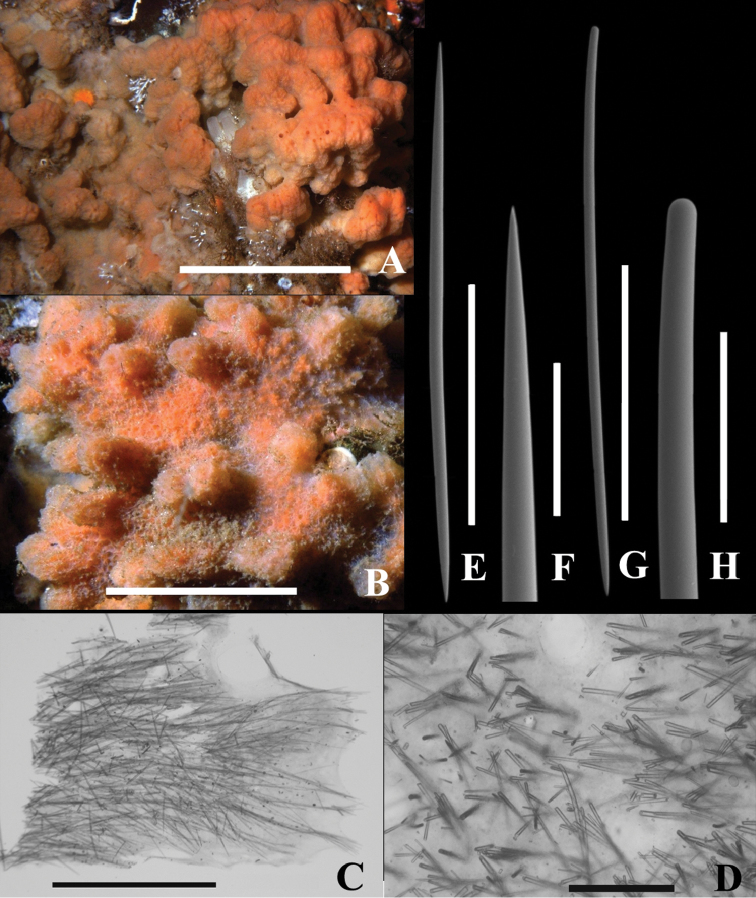
*Dragmacidon kishinensis* sp. n. **A** Holotype **B–H** Paratype **A** KML1111, in situ Steep I., BC, scale bar approx. 10 cm **B** KML1114, in situ Texada I., scale bar approx. 5 cm **C** KML1113, long. section, periphery to right, scale bar 3 mm **D** KML1113, cross section scale bar 500 µm **E** KML1113, oxea, scale bar 500 µm **F** KML1113, oxea tip, scale bar 100 µm **G** KML1113, style, scale bar 500 µm **H** KML1113, head of style, scale bar 100 µm.

***Macroscopic features*.** Thick, encrusting, unbranched form, 20 × 20 × 4 cm thick in holotype. Surface with abundant, small (1 mm diam.) and large (1 cm × 1–3 cm) irregular tubercles (Fig. 2A). Consistency preserved: moderately compressible but tough. Oscula numerous, flush with surface and ranging from 0.2 to 4 mm diameter. Aquiferous canals tangential with those near surface leading to oscula. Orange colour in life (Fig. 2A), light tan in alcohol.

*Microscopic features*. Ostia about 0.5 mm diam. in situ penetrate a thin surface membrane between lobes (Fig. 2B); weakly developed plumoreticulate skeleton of 50-100 um diameter fibers extend from base to surface; uni or pauci spicular cross connections (Fig. 2C); halichondroid (confused) skeleton toward the base. No specialized ectosomal skeleton and no axial skeleton.

##### Spicules.

Spicule types include straight oxeas (Fig. 2E), slightly curved styles (Fig. 2G), and strongyles. Oxea tips gradually and sharply pointed; style heads smoothly rounded, slightly narrower than main style body. Styles and oxeas mostly slightly curved or straight; a few strongly curved or sinuous. Strongyles uncommon to rare.

Table 2 lists spicule dimensions.

Oxea and styles in about equal numbers, and of equivalent length. A second category of styles (styles II), while of comparable length to style 1, much thinner. Also much less abundant in two specimens and absent in eight other specimens. Strongyles of comparable width to oxeas and style I, but shorter; few in number in seven specimens and absent in three other specimens. No loose raphids or trichodragmas observed.

**Table 2.** Spicule measurements of six specimens of *Dragmacidon kishinensis* sp. n. examined in detail N=50 unless indicated.

**Accession No.**	**Location**	**Sponge Tissue**	**Styles 1**	
			**L (µm)**	**W (µm)**
KML1113	Limestone I., BC		493–(916)–1300	11–(16.1)–28.6
KML1111	Steep I., BC		550–(1037)–1960	7.4–(18.0)–33
KML1112	Copper Cliffs, BC		630–(851)–1100	11–(19.6)–30.8
KML1114	Texada I, BC		520–(955)–1285	11–(14.5)–28.6
KML1116	Vivian I., BC	Ectosome	330–(738)–1060	11–(16.9)–26.1
KML1116	Vivian I., BC	Choanosome	580–(872)–1180	6.6–(15.7)–24.2
KML1121 Specimen A	Rennell Sound, BC	Ectosome	300–(591)–970	8.8–(16)–26.4
KML1121 Specimen A	Rennell Sound, BC	Choanosome	460–(809)–1150	8.8–(14.7)–22
KML1121 Specimen B	Rennell Sound BC	Ectosome	550–(930)–1238	8.8–(13.8)–19.8
KML1121 Specimen B	Rennell Sound BC	Choanosome	530–(910)–1060	4.4–(11.9)–17.6
KML1113	Limestone I., BC		580–(857)–1200	2.2–(6.1)–11
KML1111	Steep I., BC		500–(816)–1050 (n=19)	2.2–(7.4)–11 (n=19)
KML1112	Copper Cliffs, BC		n=0	
KML1114	Texada I, BC		n=0	
KML1116	Vivian I., BC	Ectosome	n=0	
KML1116	Vivian I., BC	Choanosome	n=0	
KML1121 Specimen A	Rennell Sound, BC	Ectosome	n=0	
KML1121 Specimen A	Rennell Sound, BC	Choanosome	n=0	
KML1121 Specimen B	Rennell Sound BC	Ectosome	n=0	
KML1121 Specimen B	Rennell Sound BC	Choanosome	n=0	
KML1113	Limestone I., BC		690–(1033)–1300	14.4–(20.8)–30.8
KML1111	Steep I., BC		904–(1200)–1593	12.4–(27.3)–42.2
KML1112	Copper Cliffs, BC		670–(983)–1214	15.4–(28.1)–46.2
KML1114	Texada I, BC		670–(1029)–1380	15.4–(24.2)–33
KML1116	Vivian I., BC	Ectosome	480–(890)–1200	15.4–(27.1)–37.4
KML1116	Vivian I., BC	Choanosome	780–(1021)–1238	13.2–(25.1)–35.2
KML1121 Specimen A	Rennell Sound, BC	Ectosome	390–(701)–1010	11–(21.9)–35.2
KML1121 Specimen A	Rennell Sound, BC	Choanosome	440–(966)–1285	15.4–(26.9)–33
KML1121 Specimen B	Rennell Sound BC	Ectosome	810–(1096)–1380	8.8–(19.3)–26.4
KML1121 Specimen B	Rennell Sound BC	Choanosome	500–(1006)–1285	3.3–(15.2)–26.4
KML1113	Limestone I., BC		520–(707)–800 (n=3)	22–(26.4)–33 (n=3)
KML1111	Steep I., BC		n=0	
KML1112	Copper Cliffs, BC		580–(660)–770 (n=3)	15.4–(23.5)–28.6 (n=3)
KML1114	Texada I, BC		580–(750)–900 (n=4)	15.4–(18.2)–22 (n=4)
KML1116	Vivian I., BC	Ectosome	220–(622)–900 (n=14)	15.4–(31.1)–44 (n=14)
KML1116	Vivian I., BC	Choanosome	570–(735)–900 (n=2)	22–(27.5)–33 (n=2)
KML1121 Specimen A	Rennell Sound, BC	Ectosome	210–(399)–730 (n=19)	17.6–(26.2)35.2 (n=19)
KML1121 Specimen A	Rennell Sound, BC	Choanosome	170–(467)–690 (n=6)	19.8–(26.4)–35.2 (n=6)
KML1121 Specimen B	Rennell Sound BC	Ectosome	n=0	
KML1121 Specimen B	Rennell Sound BC	Choanosome	n=0	

##### Remarks.

The paucity of thin styles suggests that these are developmental stages. The strongyles may be anomalies or, alternatively, may be associated with one area of the sponge such as the oscula or the area of attachment to the substrate.

The choanosomal skeleton of *Dragmacidon kishinensis* sp. n. is only weakly plumose. In our material the linear tracts are also less dense than in the type species *Dragmacidon agariciformis* (Dendy, 1905). In other respects it fits the diagnosis for *Dragmacidon* which includes species that are thickly encrusting, the surface with tubercles, ectosome without a specialized skeleton, and skeleton not differentiated into an axial or extra-axial region. It also has both oxeas and styles of similar form and in similar numbers.

It does not fit the diagnosis for *Axinyssa* where the choanosomal skeleton is confused; and where the spicules may be oxeas, strongyloxeas or stylote modifications of oxeas (Erpenbeck and Van Soest 2002)

Twenty-six species of *Dragmacidon* are presently recognized (van Soest et al. 2005: World Porifera Database, accessed February 2013).

Eight species can be excluded from being conspecific based on their having trichodragmas which are lacking in *Dragmacidon kishinensis* sp. n. All but two of the remaining species without trichodragmas have oxeas and styles which are at least 50% shorter than those in *Dragmacidon kishinensis* sp. n. The first exception is *Dragmacidon oxeon* which has styles and oxeas only slightly shorter than those in *Dragmacidon kishinensis* sp. n.; however, it differs from *Dragmacidon kishinensis* sp. n. in having a well developed detachable membrane. The second exception is *Dragmacidon egregium* (Ridley, 1881) which has two classes of styles, one 650–900 µm in length but the other as well as the oxeas only up to 400–450 µm in length.

The weakly developed skeleton is similar to that in *Dragmacidon grayi* (Wells & Wells in Wells et al. 1960) as described by Alvarez et al. (1998).

*Dragmacidon kishinensis* sp. n. shows some similarities to species of *Axinyssa* (Halichondridae) including a disorganized skeleton with, in some species, vaguely ascending vertical tracts toward the periphery. Spicules include oxeas and/or strongyloxeas, here considered to have a fusiform shaft which is pointed at one end and rounded at the other. *Dragmacidon kishinensis* sp. n. spicules consist of oxeas and styles, the latter are isodiametric rather than being fusiform. There are 28 described species of *Axinyssa* (van Soest et al. consulted August 2013). They are nearly all tropical.

##### Conclusions.

Based on the comparisons listed in Table 3, our *Dragmacidon* is a new species. The combination of spicule types and sizes in *Dragmacidon kishinensis* sp. n. do not match any other *Dragmacidon* species described. The reduction of a plumoreticulate skeleton and evidence of unoriented spicules suggests a possible affinity with *Axinyssa* spp. but the latter have only oxeas or strongyloxeas while our species has both oxeas and styles as found in some *Dragmacidon* spp. Finally, we would not expect to find an *Axinyssa* sp. in the cold temperate waters of British Columbia.

**Table 3.** Compares the spicules of *Dragmacidon kishinensis* sp. n. to other species of *Dragmacidon*.

*Dragmacidon agariciforme* (Dendy, 1905), p. 186	Indian Ocean	Has trichodragmas
*Dragmacidon australe* (Bergquist, 1970), p. 20	Australia, New Zealand	Surface extremely hispid, oxeas 217–260–339, styles 320–367–406
*Dragmacidon clathriforme* (Lendenfeld, 1888), p. 82	Australia	Sponge lobate; has trichodragmas
*Dragmacidon coccineum* (Keller, 1891), p. 307	Indian Ocean	Has trichodragmas
*Dragmacidon condylia* (Hooper & Lévi, 1993), p. 1405	New Caledonia	Oxeas 208–289–360, styles same
*Dragmacidon debitusae* (Hooper & Lévi, 1993), p. 1437	New Caledonia	Oxeas 223–503 styles same, rare
*Dragmacidon decipiens* (Wiedenmayer, 1989), p. 47	Bass Str., Australia	Strongyles 542–770; oxeas styles 278–350–483
*Dragmacidon durissimum* (Dendy, 1905)	Indian Ocean	Has trichodragmas
*Dragmacidon egregium* (Ridley, 1881)	S. Chile; N. Atlantic	Ectosomal styles 230–450, oxeas 280–400, axial styles 650–900
*Dragmacidon explicatum* (Wiedenmayer, 1977), p. 159	Bahamas, N. Carolina	Styles 255–332–400 and oxeas 287–333–375
*Dragmacidon fibrosum* (Ridley & Dendy, 1886), p. 481	Str. of Magellan	No oxeas; styles 630
*Dragmacidon grayi* (Wells, Wells & Gray, 1960)	N. Carolina	Oxeas 360–460 and styles 240–300
*Dragmacidon incrustans* (Whitelegge, 1897), p. 339	Gilbert/Ellise Is., S. Pacific	Styles 200–400 and oxeas 350
*Dragmacidon lunaecharta* (Ridley & Dendy, 1886), p. 481	Cape Verde E. Atlantic, Africa	No styles and oxeas 350–400
*Dragmacidon mexicanum* (de Laubenfels, 1935), p. 6	Gulf of California	Sponge very hispid; styles 400, and oxeas 300–465
*Dragmacidon mutans* (Sarà, 1978)	Tierra del Fuego	Styles 100–220, oxeas 200
*Dragmacidon ophisclera* de Laubenfels, 1935, p. 7	Gulf of California	Styles 1200; oxeas 650 smaller; loose raphids and trichodragmas present
*Dragmacidon oxeon* (Dickinson, 1945), p. 32	Gulf of California	Easily detached dermal membrane; styles 900, oxeas 600–1150, slightly smaller than *Dragmacidon kishinensis* sp. n.
*Dragmacidon reticulatum* (Ridley & Dendy, 1886), p. 481	Gulf of California	Styles 450 and oxeas 450
*Dragmacidon sanguineum* (Burton, 1933)	Natal	Styles 140 and oxeas 211
*Dragmacidon tuberosum* (Topsent, 1928), p. 178	Boavista Is., E. Atlantic, Africa	Has trichodragmas; styles 315–420, oxeas 370–420
*Dragmacidon tumidum* (Dendy, 1897), p. 236	S. Australia	No oxeas; small styles 180

##### Bathymetric range.

One intertidal record (0.2 m above 0 m [low tide]) in a cave; otherwise 3 m to 30 m depth.

##### Zoogeographic range.

Recorded from Barkley Sound (49°N) to Rennell Sound (53°N), BC.

##### Ecology.

*Dragmacidon kishinensis* sp. n. is recorded from high wave or high current energy habitats. The tough, encrusting, non branching form would be structurally adaptive for the physical impacts of strong water movement.

## Supplementary Material

XML Treatment for Auletta krautteri

XML Treatment for Dragmacidon kishinensis
